# L’aplasie tibiale. Traitement par tibialisation de la fibula et compensation du raccourcissement par maintien du pied en équinisme à Bangui, République centrafricaine

**DOI:** 10.48327/mtsi.v5i4.2025.655

**Published:** 2025-08-07

**Authors:** Michel ONIMUS, Anselme YAFONDO

**Affiliations:** 1 Faculté de médecine de Besançon, 19 Rue Ambroise Paré, 25000 Besançon, France; 2 Centre de rééducation pour handicapés moteurs (CRHAM) Bangui, République centrafricaine; 3 CHU communautaire de Bangui, République centrafricaine

**Keywords:** Aplasie tibiale, Tibialisation, Équinisme du pied, Enfants, Bangui, République centrafricaine, Afrique subsaharienne, Tibial aplasia, Tibialization, Equinus foot, Children, Bangui, Central African Republic, Sub-Saharan Africa

## Abstract

**Objectif:**

L’absence congénitale du tibia (aplasie tibiale congénitale) entraine un handicap important pour la marche. Son traitement habituel (allongement du segment jambier ou amputation précoce puis appareillage) fait appel à des moyens techniques qui font souvent défaut dans les pays en développement. L’alternative proposée ici consiste en une tibialisation de la fibula avec égalisation des membres par conservation de l’équinisme du pied.

**Patients et méthode:**

Les dossiers de 25 enfants présentant une aplasie tibiale congénitale ont été revus. Parmi ceux-ci, 10 enfants ont été opérés par tibialisation de la fibula dans 8 cas (opérés entre 1 et 3 ans) et par amputation dans 2 cas (à 6 et 8 ans).

**Résultats:**

Avec un recul moyen de deux ans et sept mois, la tibialisation de la fibula a été obtenue dans tous les cas avec un alignement correct du segment jambier et du pied sous le genou. Deux enfants ont acquis une marche indépendante. Trois enfants ont été perdus de vue.

**Discussion:**

La prise en charge de l’aplasie tibiale congénitale doit tenir compte du contexte socio-économique local. Bien que le recul de la série présentée soit peu important, le protocole utilisé a l’avantage de la simplicité et d’être bien accepté par les familles.

**Conclusion:**

Le traitement conservateur de l’aplasie tibiale par tibialisation de la fibula avec conservation de l’équinisme pour compenser le raccourcissement est un traitement simple, qui tient compte des impératifs socioéconomiques locaux et qui évite les contraintes et les coûts des protocoles modernes. Il est de plus toujours accepté par les familles.

## Abstract (en)

**Objective:**

Congenital absence of the tibia (congenital tibial aplasia) causes significant disability when walking. The usual treatment, which involves lengthening the lower leg or early amputation followed by prosthetic fitting, requires technical resources that are often lacking in developing countries. The proposed alternative consists of tibialization of the fibula with limb lengthening and by preservation of the foot equinismus.

**Patients and method:**

The records of 25 children with congenital tibial aplasia were reviewed. Ten of these children underwent tibialization of the fibula (operated between one and three years of age) and amputation (at six and eight years of age) in two cases.

**Results:**

With a mean follow-up period of two years and seven months, correct alignment of the lower leg and foot below the knee was achieved in all cases of tibialization of the fibula. Two children achieved independent walking. Three children were lost for follow-up.

**Discussion:**

The management of congenital tibial aplasia must consider the local socioeconomic context. Although the follow-up period in this study is short, the protocol is simple and well-accepted by families.

**Conclusion:**

Conservative treatment of tibial aplasia through tibialization of the fibula with equinus preservation to compensate the shortening is simple and takes local socioeconomic constraints into account, while avoiding the limitations and costs of modern protocols. Furthermore, it is always well accepted by families.

## Introduction

L’hémimélie tibiale, ou aplasie congénitale du tibia, est de traitement difficile: on conseille habituellement une amputation précoce avec appareillage par prothèse, si possible autour de l’âge de la marche. Cependant dans certaines situations, et notamment en fonction de traditions culturelles, l’amputation est mal acceptée; de plus, l’appareillage induit des coûts qui peuvent être difficiles à supporter par les familles. L’alternative est un traitement conservateur, qui impose une égalisation des membres par allongement du segment jambier, mais il s’agit de techniques longues, nécessitant un matériel spécifique et demandant une très bonne coopération de la part de la famille, conditions qui ne sont pas toujours retrouvées. Les inconvénients de ces deux protocoles nous ont poussés à proposer un traitement conservateur plus simple, associant une tibialisation de la fibula et une compensation du raccourcissement du segment jambier par la conservation de l’équinisme du pied.

## Patients et méthode

Ce travail a été réalisé dans le cadre des activités de l’association des Amis comtois des missions centrafricaines (ACMC) qui organise depuis 41 ans des missions chirurgicales de prise en charge d’enfants handicapés en République centrafricaine et qui fournit le consommable nécessaire aux interventions. L’activité chirurgicale se déroule dans les services de chirurgie infantile et de traumatologie du Centre national hospitalo-universitaire (CNHU) de Bangui. Les consultations ainsi que l’activité de rééducation et d’appareillage ont été réalisées dans le Centre de rééducation pour handicapés moteurs (CRHAM) de Bangui, structure diocésaine. Les enfants ont été soit évacués de province sur le CRHAM, soit amenés spontanément au CRHAM par les familles, soit référés au CRHAM par les chirurgiens ou médecins du Complexe pédiatrique du CNHU de Bangui. L’intervention a été proposée aux familles lorsque la tibialisation de la fibula paraissait réalisable, c’est-à-dire lorsqu’un moignon tibial était présent cliniquement. Elle a été acceptée dans tous les cas. Compte tenu de la précarité de la situation sociale de la plupart des familles, l’objectif était d’éviter un appareillage par prothèse toujours coûteux. Vingt-cinq enfants présentant une aplasie tibiale ont été vus à un âge moyen de 4 ans et ont fait l’objet de ce travail. Parmi ceux-ci, 17 ont été vus avant l’âge de 4 ans, 6 entre 4 et 9 ans, et 2 patients ont été vus respectivement à 15 ans et 28 ans. L’aplasie était bilatérale dans 8 cas et unilatérale dans 17 cas, 9 fois du côté droit et 8 fois du côté gauche. Un bilan radiographique initial n’a pu être réalisé que dans huit cas, rendant difficile le classement de quelques cas. Des radiographies en cours de traitement ont pu être obtenues dans tous les cas opérés. L’aplasie a été classée selon Kalamchi [11] comme totale dans 11 cas (type I) chez 7 enfants, partielle dans 19 cas (type II) chez 16 enfants, avec présence d’une extrémité supérieure du tibia de longueur variable, et de type III avec diastasis tibio-fibulaire inférieur dans 3 cas. Dans 5 cas, l’existence d’une épiphyse tibiale supérieure a été difficile à affirmer; elle a été suspectée lorsque le genou a paru stable à l’examen et en l’absence de flexum irréductible du genou.

## Protocole opératoire

La tibialisation de la fibula se fait en deux temps opératoires. Le premier temps consiste en une tibialisation basse de la fibula. Par une incision antérieure on aborde l’extrémité inférieure de la fibula et on ouvre la capsule solidarisant la fibula au talus. Il faut sectionner cette capsule en totalité et la dissection doit être faite au ras de l’os et du cartilage pour éviter de léser les éléments tendineux et vasculo-nerveux. Du fait des rétractions médiales, il est impossible à ce stade de repositionner le talus sous la fibula et il faut toujours raccourcir le squelette, en aplanissant le dôme du talus et en reséquant l’extrémité inférieure de la fibula. Le noyau épiphysaire inférieur de la fibula n’est pas encore ossifié et il faut réséquer dans la maquette cartilagineuse jusqu’à ce qu’on puisse amener le talus sous la fibula. Il est nécessaire d’allonger le tendon d’Achille; les parties molles postéro-médiales sont toujours très rétractées, et la conservation de l’équinisme facilite le positionnement du talus sous la fibula. La persistance d’un léger varus du pied peut être tolérée car il sera corrigé secondairement lors de la tibialisation haute de la fibula. Idéalement, la fixation se fait par une broche introduite en va-etvient dans le talus et le calcanéum et remontant dans la fibula. Le jeune âge des enfants explique probablement que la fibula se tibialise dès les premiers mois postopératoires en s’élargissant et en s’épaississant, malgré l’absence de mise en charge. Après l’intervention un plâtre cruro-pédieux est appliqué pour trois mois.

Le deuxième temps opératoire est réalisé trois à quatre mois après le premier temps. Il consiste en une tibialisation haute de la fibula. Par une incision externe il faut sectionner la fibula à environ 1 cm au-dessus de l’extrémité inférieure du tibia, puis transposer la fibula sous le moignon tibial par une seconde incision antérieure, en réséquant l’extrémité du tibia de façon à ouvrir le canal médullaire. La fixation se fait également par une broche centromédullaire introduite en va-et-vient. Un nouveau plâtre cruro-pédieux est appliqué pour 3 mois, suivi d’une botte plâtrée permettant la marche, pour encore deux à trois mois.

## Résultats

**Figure 1:**
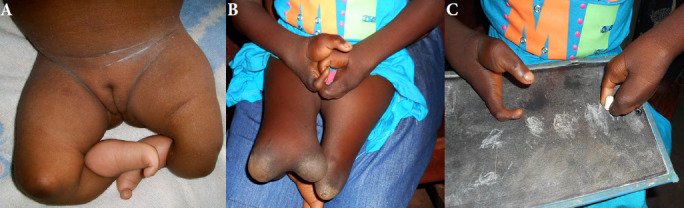
Syndrome de Gollop-Wolfgang. (A) L’enfant présente une aplasie tibiale bilatérale avec oligodactylie au niveau des deux pieds. (B) Il existe une duplication fémorale inférieure à droite. (C) Au niveau des mains il existe une malformation en pince de homard bilatérale. La fonction est cependant très bonne avec une bonne préhension

Des malformations associées à une aplasie tibiale bilatérale ont été dépistées par l’examen clinique dans un cas évoquant un syndrome de Gollop-Wolfgang [21] avec duplication fémorale inférieure, oligodactylie au niveau des pieds et mains en pince de homard (Fig. 1).

**Figure 2:**
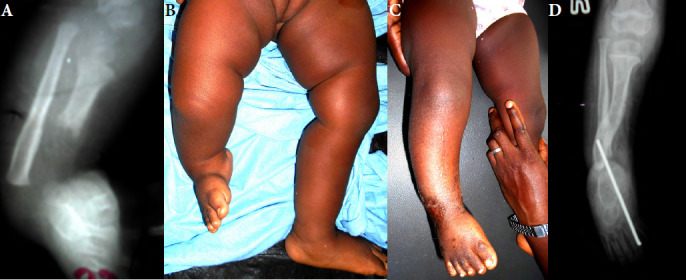
Aplasie partielle du tibia droit. L’enfant est vue à 12 mois; une tibialisation basse est réalisée à 15 mois, suivie d’une tibialisation haute à 24 mois. (A, B) Aspect clinique et radiographique pré-opératoire. (C, D) Aspect après tibialisation haute et basse

**Figure 3:**
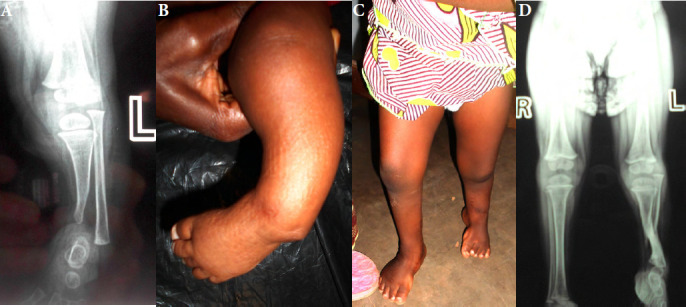
Enfant vue à 5 mois. Tibialisation basse de la fibula à 12 mois, puis tibialisation haute à 15 mois. À 24 mois la marche est autonome avec un raccourcissement d’environ 5 cm compensé par l’équinisme du pied. (A, B) Aspect à l’âge de 5 mois. (C, D) Résultat à l’âge de 2 ans

Neuf enfants ont été opérés entre 2009 et 2023. Deux d’entre eux ont été opérés à 6 et 8 ans par amputation. Ils ont été revus avec un recul de deux ans et de cinq ans. Sept enfants ont été opérés entre six mois et deux ans, par tibialisation de la fibula (Fig. 2). Trois d’entre eux ont été perdus de vue. Dans un cas, le recul est insuffisant pour juger du résultat. Enfin, trois cas ont pu être revus avec un recul de six mois, deux ans et deux ans. Une marche indépendante était acquise dans les trois cas (Fig. 3). Il n’a pas été observé d’échec de la tibialisation de la fibula; dans deux cas un varus résiduel du pied a nécessité une reprise chirurgicale (Tableau I).

**Tableau I:** Description de la série

Nom	Age	Siège	Type	Radio	Lésions associées	Traitement	Recul	Résultat
1. BF	15 ans	Bilatéral	D type I - G type II	0	Bifidité fémur D	Néant	0	Marche sur les genoux
2. KB	5 ans	Unilatéral G	Type III	+	Néant	5 ans: allongement Achille	1 an	Marche acquise
3. BD	5 ans	Unilatéral G	Type II	0	Aplasie fibula D	Néant	0	Marche à quatre pattes
4. BD	7 ans	Bilatéral	D type III - G type II	0	Néant	Néant	0	Marche à quatre pattes
5. YM	6 ans	Unilatéral G	Type I	0	Néant	8 ans: amputation	2 ans	Marche avec prothèse
6. YD	3 ans	Unilatéral G	Type I	0	Néant	Néant	0	Marche avec orthèse
7. AI	9 ans	Bilatéral	D type II - G type II	0	Néant	Néant	0	Marche à quatre pattes
8. YD	3 ans	Unilatéral G	Type II	0	Néant	Néant	0	Marche avec béquilles
9. YA	6 mois	Bilatéral	D type I - G type I	0	Néant	Néant	0	Marche à quatre pattes
10. AA	5 mois	Unilatéral G	Type II	+	Pied bot varus équin (PBVE) D	6 mois: tibialisation basse 12 mois: tibialisation haute 15 mois: reprise basse	2 ans	Marche acquise
11. KA	2 ans	Unilatéral D	Type II	0	Néant	Néant	0	Marche avec béquilles
12. A R	1 mois	Bilatéral	D type I - G type I	0	Pince de homard - Hypoplasie des pieds	Néant	0	Marche sur les genoux
13. Y A	2 ans	Unilatéral D	Type II	0	Hallux valgus D	2 ans: tibialisation basse	0	Perdu de vue
14. BG	4 ans	Unilatéral G	Type II	0	Néant	6 ans: amputation	5 ans	Marche avec prothèse
15. WB	1 an	Unilatéral D	Type II	+	Néant	1 an: tibialisation basse 15 mois: tibialisation haute	6 mois	Marche acquise
16. MB	4 ans	Unilatéral D	Type II	0	Néant	Néant		Perdu de vue
17. DF	28 ans	Unilatéral D	Type II	0	Néant	Néant	0	Marche avec béquilles
18. BF	2 ans	Unilatéral D	Type II	0	Néant	Néant	0	Perdu de vue
19. KJ	9 ans	Unilatéral D	Type III	0	Néant	Néant	0	Marche
20. MM	1 an	Unilatéral D	Type II	+	Néant	14 mois: tibialisation basse	0	Perdu de vue
21. ZM	2 mois	Bilatéral	D type I G type I	0	Néant	Néant	0	Perdu de vue
22. MM	6 mois	Bilatéral	D type II - G type II	+	Néant	9 mois: tibialisation basse G 12 mois: tibialisation haute G, tibialisation basse D 15 mois: tibialisation haute D, reprise basse G	0	Marche non acquise
23. NT	1 mois	Unilatéral G	Type II	+	Néant	6 mois: tibialisation basse	0	Perdu de vue
24. TW	1 an	Bilatéral	D type I - G type I	+	Syndactylie deux mains	Amputation proposée	0	Perdu de vue
25. GY	1 an	Unilatéral D	Type II	+	Néant	12 mois: tibialisation basse 16 mois: tibialisation haute	2 ans	Marche

## Discussion

Ce travail présente d’importantes limites liées au contexte dans lequel il a été entrepris: une radiographie n’a pu être réalisée que dans un petit nombre de cas. De plus, la radiographie peut être insuffisante en cas de type II lorsque le moignon tibial est présent mais non encore ossifié. Une échographie ou même une IRM permettraient de visualiser un moignon cartilagineux, mais ces examens n’étaient pas disponibles. Neuf patients (8 enfants et un adulte) n’ont été vus qu’une seule fois, ne permettant qu’un bilan lésionnel, mais sans prise en charge chirurgicale. Il s’agissait notamment des types I de Kalamchi, donc sans épiphyse tibiale certaine, pour lesquels il n’a pas été retenu d’indication chirurgicale. Par ailleurs, une prise en charge de longue durée est parfois mal acceptée en milieu africain, et quelques enfants ont été rapidement perdus de vue. L’absence de recul à long terme est également préjudiciable car une aggravation secondaire du raccourcissement peut se produire, notamment si le temps opératoire de tibialisation basse a comporté une résection de la zone de croissance de la fibula. Par ailleurs, les risques de récidive du varus du pied sont importants jusqu’en fin de croissance. Ainsi, dans la série de Wada *et al.* [20], 15 pieds sur un total de 19 ont dû être réopérés pour récidive. Le protocole présenté ici est donc une étude préliminaire, qui demande à être validée par une revue des résultats avec un recul suffisant.

L’hémimélie tibiale est une malformation considérée comme rare dans la littérature: sa fréquence serait de l’ordre d’un cas sur 1000 000 naissances. Elle est fréquemment associée à d’autres malformations qui ne seront pas envisagées dans ce travail. Différentes classifications ont été proposées. La classification de Jones [10] modifiée par Kalamchi [11] a l’avantage d’être simple et de pouvoir être utilisée pratiquement au vu du seul examen clinique.

**Figure 4:**
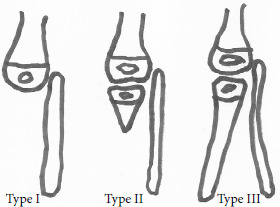
Classification de Kalamchi

Kalamchi distingue 3 types (Fig. 4):

- Le type I (type IA de Jones) est caractérisé par une absence complète du tibia, avec absence de tendon rotulien et flexum irréductible du genou. Nous l’avons retrouvé chez 7 enfants, soit 28%. Ce chiffre est probablement sousestimé car dans la littérature sa fréquence est variable, de 30% jusqu’à 60% des cas dans la série de Clinton qui porte sur 125 cas d’aplasie tibiale [5].
- Le type II (types IB et II de Jones) est caractérisé par la présence d’une épiphyse tibiale proximale, de longueur variable, parfois simple maquette cartilagineuse, parfois noyau osseux présent à la naissance. Le diagnostic peut être difficile à la naissance car une épiphyse tibiale cartilagineuse est parfois de petit volume. Il faut s’attacher à rechercher la présence de la tubérosité tibiale et d’une extension active du genou en faveur de la présence d’un quadriceps. Le flexum de genou est modéré et réductible. Nous avons retrouvé un type II chez 19 enfants, soit 64% des cas, chiffre sans doute surestimé en raison de l’absence de moyens d’exploration fiables. La fréquence du type II est également variable dans la littérature, allant de 20% des cas dans la série de Clinton [5] à environ 40% des cas dans la méta-analyse de Sola [18] portant sur 131 cas.
- Le type III est plus rare (type IV de Jones). Il est caractérisé par une aplasie partielle basse du tibia avec diastasis tibio-fibulaire inférieur. Nous avons retrouvé 2 cas, soit 8%.

La demande principale des parents d’un enfant porteur d’une aplasie tibiale concerne l’acquisition de la marche. Spontanément, celle-ci est généralement acquise avec appui monopodal avec l’aide d’une béquille ou d’un simple bâton, mais une marche bipodale libère les mains et donne une meilleure autonomie à l’enfant.

Dans la littérature, la désarticulation au niveau du genou avec appareillage par prothèse est considérée comme le meilleur traitement, apportant des résultats supérieurs au traitement conservateur, tant en termes de durée et de contraintes du traitement que de résultats fonctionnels, en particulier dans le type I de Kalamchi [7,18]. Cependant, l’amputation implique la fabrication d’une prothèse, qu’il faudra renouveler tout au long de la croissance de l’enfant. Or dans des milieux défavorisés ou éloignés, un atelier d’appareillage compétent n’est pas toujours disponible à proximité du lieu d’habitation de l’enfant. Deux enfants de notre série venaient de localités de province situées à distance et un appareillage par prothèse n’était pas envisageable pour eux. Par ailleurs, l’appareillage induit des coûts financiers que les familles ne peuvent pas ou ne veulent pas supporter. À Bangui, l’association Handicap international a créé le Centre national d’appareillage, mais le coût d’une prothèse est difficile à faire accepter et surtout son renouvellement durant la croissance est loin d’être assuré. Enfin, et ceci est un important élément à prendre en compte, l’amputation est en règle générale très mal acceptée dans des milieux traditionnels. Dans l’étude de Eamsobhana *et al.* [6] faite sur six cas en milieu thaï, le traitement conservateur était préféré à l’amputation dans tous les cas, même au prix de multiples interventions y compris en cas de résultats médiocres. Dans la série de 24 patients d’origine indienne rapportée par Kumar Sahoo [12], l’amputation n’a été acceptée que dans un cas. Dans un de nos cas (observation N° 24) l’amputation a été proposée aux parents car l’épiphyse tibiale supérieure paraissait absente, mais elle a été refusée et l’enfant a été perdu de vue.

Une alternative à l’amputation est la conservation de la jambe avec tibialisation de la fibula, technique proposée par Huntington dès 1905 [9] pour la reconstruction du tibia après perte de substance d’origine traumatique ou infectieuse, ou encore après résection pour tumeur [14,19]. En cas d’aplasie tibiale congénitale de type II, de nombreux auteurs ont proposé une tibialisation haute de la fibula [11,16], geste qui permet de stabiliser la fibula et de réaligner le membre inférieur, en y associant une prothèse après amputation du pied selon la technique de Syme ou Boyd. Cependant, l’appareillage pose les mêmes problèmes matériels que l’amputation ou la désarticulation au genou. Pour éviter une prothèse, l’alternative est de réaliser un allongement de la fibula par le matériel d’Ilizarov ou ses dérivés, associé à une synostose tibio-fibulaire en haut et un recentrage du talus sous la fibula avec une synostose talo-fibulaire en bas, et de bons résultats de cette technique ont été publiés [14,15]. Cependant, les techniques d’allongement osseux sont des programmes lourds et longs, qui nécessitent des instrumentations pas toujours disponibles, qui demandent beaucoup de temps et surtout une très bonne coopération de la part des familles, conditions qui ne sont pas toujours présentes.

Lorsque ces conditions ne sont pas réunies et que le milieu ne s’y prête pas, il est donc préférable de ne pas se référer à des protocoles impossibles ou difficiles à mettre en œuvre. Dans ces cas, le raccourcissement du segment jambier peut être simplement compensé par la conservation de l’équinisme du pied, permettant l’appui au sol en digitigrade. Cet appui ne permet pas un déroulement normal du pas, mais une marche autonome peut être acquise. Les interventions ne demandent pas de matériel spécifique en dehors d’instruments fins. Elles sont réalisées sous hémostase par garrot, rendant le geste peu agressif. Cependant, la tibialisation de la fibula avec conservation de l’équinisme du pied n’est pas réalisable dans tous les cas: le pied ne doit pas être malformé, de façon à permettre un appui digitigrade correct. Une épiphyse tibiale supérieure doit être présente, ce qui élimine les cas d’aplasie de type I de Kalamchi car en l’absence d’épiphyse tibiale supérieure, la fibula ne peut être que transposée sous les condyles fémoraux; c’est l’opération de Brown [2,3] qui ne donne jamais un genou stable [1,13]. Par ailleurs, le flexum du genou présent dans le type I est de correction très difficile et récidivant. Les résultats satisfaisants observés après opération de Brown ont le plus souvent été obtenus après désarticulation du genou et appareillage [4,17]. Il faut donc réserver l’indication de tibialisation aux types II de Kalamchi avec présence d’une extrémité supérieure du tibia et d’un quadriceps fonctionnel.

L’objectif étant de permettre à l’enfant de commencer à marcher le plus possible à l’âge normal, la tibialisation de la fibula doit être réalisée précocement, idéalement autour de l’âge d’un an. À cet âge, la maquette cartilagineuse est encore très présente et lors du premier temps chirurgical (tibialisation basse de la fibula) on met en contact des surfaces qui sont plus cartilagineuses qu’osseuses. Cependant, la stabilité du pied sous la fibula est bonne après ablation de la broche et l’existence du cartilage semble permettre une poursuite de la croissance de la fibula.

Enfin, le raccourcissement en fin de croissance ne doit pas être supérieur à 10 ou au maximum 15 cm, car au-delà l’inégalité ne peut pas être compensée par l’équinisme. Héchard et Carlioz [8] ont montré qu’en cas de malformation congénitale, le pourcentage d’inhibition de croissance reste stable tout au long de la croissance. En calculant à la naissance le pourcentage de raccourcissement, on peut donc prévoir l’importance de l’inégalité qui sera retrouvée à maturité osseuse. En revanche, en cas d’aplasie tibiale bilatérale, présente dans 25% des cas, le problème de l’inégalité de longueur ne se pose pas et la tibialisation bilatérale de la fibula peut être proposée sans arrière-pensée.

## Conclusion

L’hémimélie tibiale, ou aplasie congénitale du tibia, est une malformation handicapante et de traitement difficile. En l’absence de prise en charge, l’enfant se déplace à quatre pattes durant les premières années, puis debout à l’aide d’une béquille. Un traitement conservateur par tibialisation de la fibula avec compensation du raccourcissement du segment jambier par la conservation de l’équinisme du pied peut être proposé lorsqu’une ébauche tibiale est présente. Ce traitement est simple et il peut être réalisé même dans des conditions sommaires. Il permet une marche sur le pied en équinisme et même s’il ne permet pas un déroulement normal du pas, il permet à l’enfant d’acquérir une marche autonome, sans aide ni béquille. Il s’agit d’une technique de nécessité, très éloignée des techniques modernes, mais qui offre l’avantage de s’affranchir des contraintes culturelles souvent présentes et de diminuer les coûts élevés d’une chirurgie d’allongement ou d’un appareillage prothétique dans les pays en développement.

## Consentement des parents

La technique chirurgicale utilisée n’est pas innovante en ce sens qu’elle a déjà été décrite dans la littérature. De ce fait, l’avis d’un comité d’éthique n’a pas été sollicité. Les parents ont été informés du déroulement du protocole chirurgical et de la nécessité de plusieurs opérations.

## Source de financement

Ce travail n’a bénéficié d’aucune source de financement.

## Contributions des auteurs

AY: révision des dossiers et relecture du texte MO: rédaction de l’article

## Conflits d’intérêts

Les auteurs ne déclarent aucun conflit d’intérêts.
