# Supplementary material for: Tibial aplasia. Treatment by tibialization of the fibula and compensation for shortening by holding the foot in equinus position in Bangui, Central African Republic
Source: Med Trop Sante Int. 2025 Aug 7;5(4):mtsi.v5i4.2025.655. [Article in French] doi: 10.48327/mtsi.v5i4.2025.655 (PMC12948360; doi:10.48327/mtsi.v5i4.2025.655)
Supplement: Related texts

# Research article: Tibial aplasia. Treatment by tibialization of the fibula and compensation for shortening by holding the foot in equinus position in Bangui, Central African Republic

## Introduction

Tibial hemimelia, also known as congenital aplasia of the tibia, is a challenging condition to treat. Early amputation followed by prosthetic fitting is typically recommended, ideally around the age when a child begins to walk. However, amputation is not always accepted, particularly depending on cultural traditions, and prosthetic fitting can be costly for families. An alternative is conservative treatment, which involves lengthening the lower leg to equalize the limbs. However, these techniques are lengthy and require specific equipment and excellent cooperation from the family, conditions that are not always met. Due to the disadvantages of these two protocols, we propose a simpler conservative treatment combining tibialization of the fibula and compensation of the shortening of the leg by preserving the equinus foot deformity.

## Patients et method

This work was carried out as part of the Association des Amis Comtois des Missions Centrafricaines’ (ACMC) activities. For 41 years, ACMC has organized surgical missions to care for disabled children in the Central African Republic and provided the necessary supplies for the operations. The surgeries are performed in the pediatric surgery and trauma departments of the National University Hospital Center (CNHU) in Bangui. Consultations, rehabilitation, and fitting of orthopedic devices took place at the Diocesan Rehabilitation Center for the Physically Disabled (CRHAM) in Bangui. The children were either evacuated from the provinces to the CRHAM, brought to the CRHAM spontaneously by their families, or referred to the CRHAM by surgeons or doctors from the pediatric complex of the CNHU in Bangui. The procedure was offered to families when tibialization of the fibula appeared feasible, i.e., when a tibial stump was clinically present. It was accepted in all cases. Given the precarious social situation of most families, the aim was to avoid the use of prostheses, which are always expensive.

A total of 25 children with tibial aplasia were included in this study, with an average age of 4 years. Seventeen of the children were seen before the age of 4, 6 were seen between the ages of 4 and 9, and 2 were seen at ages 15 and 28, respectively. Aplasia was bilateral in eight cases and unilateral in 17 cases. It occurred on the right side in nine cases and on the left side in eight cases. Only an initial radiographic assessment could be performed in eight cases, which made it difficult to classify some cases. Radiographs were obtained during treatment for all cases that underwent surgery. According to Kalamchi, [11], aplasia was classified as total (type I) in 11 cases (7 children), partial (type II) in 19 cases (16 children) with a tibial upper extremity of variable length, and type III with lower tibiofibular diastasis in three cases. The existence of an upper tibial epiphysis was difficult to confirm in five cases; it was suspected when the knee appeared stable upon examination and there was no irreducible flexion of the knee.

## Surgical Protocol

Tibialization of the fibula is performed in two stages. The first stage involves low tibialization of the fibula. An anterior incision is made to access the lower end of the fibula, and the capsule connecting the fibula to the talus is opened. The capsule must be completely severed, and dissection must be performed close to the bone and cartilage to avoid damaging tendons and neurovascular structures. Due to medial retractions, it is impossible to reposition the talus under the fibula at this stage, so the skeleton has to be shortened by flattening the dome of the talus and resecting the lower end of the fibula. Since the lower epiphyseal nucleus of the fibula has not ossified yet, the resection must be performed in the cartilaginous model until the talus can be positioned under the fibula. The Achilles tendon must be lengthened. Because the posteromedial soft tissues are retracted, preservation of equinus facilitates positioning the talus under the fibula. A slight varus of the foot can be tolerated as it will be corrected during high tibialization of the fibula. Ideally, fixation is achieved by inserting a pin back and forth through the talus and calcaneus and into the fibula. The young age of the patients likely explains why the fibula tibializes during the first few postoperative months, despite the absence of weight bearing, by widening and thickening. After the operation, a crutched cast is applied for three months.

The second stage of the operation is performed three to four months after the first stage. It consists of high tibialization of the fibula. A lateral incision is made, and the fibula is sectioned approximately 1 cm above the lower end of the tibia. Then, it is transposed under the tibial stump through a second anterior incision. The end of the tibia is resected to open the medullary canal. Fixation is achieved by inserting a centromedullary pin back and forth. A new crural cast is applied for three months, followed by a plaster boot that allows walking for an additional two-to three-month duration.

## Results

Malformations associated with bilateral tibial aplasia were detected by clinical examination in one case suggestive of Gollop-Wolfgang syndrome [21], which is characterized by lower femoral duplication, oligodactyly of the feet, and lobster claw hands (Fig. 1).

**Figure 1:** Gollop-Wolfgang syndrome. (A) The child presents with bilateral tibial aplasia with oligodactyly in both feet. (B) There is a lower femoral duplication on the right side. (C) There is a bilateral lobster claw deformity in the hands. However, function is very good with good grip

Nine children underwent surgery between 2009 and 2023. Two of them underwent amputation at the ages of six and eight. They were reviewed after two and five years, respectively. Seven children underwent surgery between the ages of six months and two years with tibialization of the fibula (Fig. 2). Three of them were lost for follow-up. In one case, the follow-up period was insufficient to assess the outcome. Finally, three cases were reviewed after six months, two years, and two years respectively. All three of them achieved independent walking (Fig. 3). There was no failure of tibialization of the fibula. In two cases, residual varus of the foot required revision of the surgery (Table I).

**Figure 2:** Partial aplasia of the right tibia. The child is seen at 12 months; low tibialization is performed at 15 months, followed by high tibialization at 24 months. (A,B) Preoperative clinical and radiographic appearance. (C,D) Appearance after high and low tibialization

**Figure 3:** Child seen at 5 months. Low tibialization of the fibula at 12 months, then high tibialization at 15 months. At 24 months, the child is walking independently with a shortening of approximately 5 cm compensated by equinus of the foot. (A,B) Appearance at 5 months. (C,D) Result at 2 years

**Table I:** Series description

Name	Age	Location	Type	Radio	Associated lesions	Treatmentt	Follow-up	Result
1. BF	15 years	Bilateral	R type I - L type II	0	Femur bifurcation R	None	0	Kneeling
2. KB	5 years	Unilatéral L	Type III	+	None	5 years: Achilles lengthening	1 year	Acquired walking
3. BD	5 years	Unilateral L	Type II	0	Fibula aplasia R	None	0	Crawling
4. BD	7 years	Bilateral	R type III - L type II	0	None	None	0	Crawling
5. YM	6 years	Unilateral L	Type I	0	None	8 years: amputation	2 years	Walking with prosthesis
6. YD	3 years	Unilateral L	Type I	0	None	None	0	Walking with orthesis
7. AI	9 years	Bilateral	R type II - L type II	0	None	None	0	Crawling
8. YD	3 years	Unilateral L	Type II	0	None	None	0	Walking with crutches
9. YA	6 months	Bilateral	R type I - L type I	0	None	None	0	Crawling
10. AA	5 months	Unilateral L	Type II	+	Congenital talipes equinovarus (CTEV) right	6 months: low tibialis 12 months: high tibialis 15 months: low revision	2 years	Acquired walking
11. KA	2 years	Unilateral R	Type II	0	None	None	0	Walking with crutches
12. A R	1 month	Bilateral	R type I - L type I	0	Lobster claw - Foot hypoplasia	None	0	Kneeling
13. Y A	2 years	Unilateral R	Type II	0	Hallux valgus R	2 years low tibialization	0	Lost for follow-up
14. BG	4 years	Unilatéral L	Type II	0	None	6 years: amputation	5 years	Walking with prosthesis
15. WB	1 year	Unilatéral R	Type II	+	None	1 year: low tibialization 15 months: high tibialization	6 months	Acquired walking
16. MB	4 years	Unilatéral R	Type II	0	None	None		Lost for follow-up
17. DF	28 years	Unilatéral R	Type II	0	None	None	0	Walking with crutches
18. BF	2 years	Unilatéral R	Type II	0	None	None	0	Lost for follow-up
19. KJ	9 years	Unilatéral R	Type III	0	None	None	0	Walking
20. MM	1 year	Unilatéral R	Type II	+	None	14 months low tibialization	0	Lost for follow-up
21. ZM	2 months	Bilateral	R type I - L type I	0	None	None	0	Lost for follow-up
22. MM	6 months	Bilateral	R type II - L type II	+	None	9 months: low tibialization L 12 months: high tibialization L, low tibialization R 15 months: high tibialization R, low revision L	0	Walk not acquired
23. NT	1 month	Unilatéral L	Type II	+	None	6 months: low tibialization	0	Lost for follow-up
24. TW	1 year	Bilateral	R type I - L type I	+	Syndactyly both hands	Proposed amputation	0	Lost for follow-up
25. GY	1 year	Unilatéral R	Type II	+	None	12 months: low tibialization 16 months: hightibialization	2 years	Walking

## Discussion

This study has significant limitations related to the context in which it was conducted. X-rays could only be performed in a limited number of cases. Additionally, X-rays may be insufficient for type II cases, when the tibial stump is present but not yet ossified. Ultrasound or MRI would allow visualization of a cartilaginous stump; however, these examinations were not available. Nine patients (eight children and one adult) were seen only once, which allowed for an assessment of the lesion but not for surgical management. These patients had Kalamchi type I lesions, meaning they did not have a definite tibial epiphysis. No surgical intervention was considered for these patients. Additionally, long-term management is sometimes poorly accepted in African settings, and some children were quickly lost for follow-up. A lack of long-term follow-up can also be detrimental because secondary worsening of the shortening may occur, particularly if the low tibialization involved resection of the fibular growth plate. Furthermore, there is a significant risk of foot varus recurrence until end of growth. In Wada *et al.’s* series [20], 15 out of 19 feet required reoperation due to recurrence. The protocol presented here is a preliminary study that needs to be validated by reviewing the results with sufficient hindsight. Tibial hemimelia is a rare malformation, that occurs at a frequency of approximately one case per million births. It is often associated with other malformations that are not the focus of this study. Various classifications have been proposed. The Jones classification [10], modified by Kalamchi [11], has the advantage of being simple and practical, based on clinical examination alone. Kalamchi distinguishes three types (Fig. 4):

**Figure 4:** Kalamchi classification

- Type I (Jones type IA): Complete absence of the tibia, absence of the patellar tendon, and irreducible flexion of the knee. We observed this condition in seven children, accounting for 28% of cases. However, this figure is likely an underestimation, as its frequency in the literature varies from 30% to 60% in Clinton’s series of 125 cases of tibial aplasia [5].
- Type II (Jones types IB and II) is characterized by the presence of a proximal tibial epiphysis of variable length. Sometimes it is a simple cartilaginous model; other times, a bone nucleus is present at birth. Diagnosis can be difficult at birth because a cartilaginous tibial epiphysis is sometimes small in volume. It is important to check for the presence of the tibial tuberosity and active knee extension, which indicates the presence of a quadriceps. Knee flexion is moderate and reducible. We found type II in 19 children, or 64% of cases. This figure is undoubtedly overestimated due to the lack of reliable investigative methods. The prevalence of type II also varies in the literature, ranging from 20% in Clinton’s [5] series to approximately 40% in Sola’s meta-analysis [18] of 131 cases.
- Type III is less frequent (Jones type IV). It is characterized by partial aplasia of the lower tibia with lower tibiofibular diastasis. We found two cases, or 8%.

The main request of parents with a child who has tibial aplasia is for their child to get walking ability. This is usually achieved spontaneously with monopodal support, such as a crutch or a simple stick. However, bipedal walking frees the hands and gives the child greater independence. According to the literature, knee disarticulation with a prosthetic fitting is considered the best treatment. It achieves better results than conservative treatment in terms of treatment duration, constraints, and functional outcomes, particularly for Kalamchi type I [7,18]. However, amputation requires the fabrication of a prosthesis that will need to be replaced during growth. But, in disadvantaged or remote areas, a competent prosthetic workshop is not always available close to the child’s home. Two children in our study came from remote rural areas, so prosthetic fitting was not an option for them. Furthermore, prosthetic fitting involves financial costs that families cannot or are unwilling to bear. In Bangui, Handicap International set up the National Fitting Center, but the cost of a prosthesis is prohibitive, and its replacement during growth is far from guaranteed. Finally, amputation is generally very poorly accepted in traditional communities, which is an important factor to consider. In a study of six cases in Thailand, Eamsobhana *et al.* [6] found that conservative treatment was preferred to amputation in all cases, even at the cost of multiple interventions, including cases with poor results. In a series of 24 patients of Indian origin reported by Kumar Sahoo, [12] amputation was accepted in one case only. In one of our cases (observation no. 24), amputation was proposed to the parents because the upper tibial epiphysis appeared absent. However, the parents refused, and the child was lost for follow-up.

An alternative to amputation is leg preservation with tiibialization of the fibula, a technique proposed by Huntington in 1905 [9] for tibial reconstruction after traumatic or infectious bone loss, or after tumor resection [14,19]. For type II congenital tibial aplasia, several authors have recommended high tibialization of the fibula [11,16]. This procedure stabilizes the fibula and realigns the lower limb. It is combined with a prosthesis after amputation of the foot using the Syme or Boyd technique. However, a prosthesis poses the same problems as amputation or knee disarticulation.

One alternative to prosthesis is to lengthen the fibula with Ilizarov equipment or derivatives, combined with tibiofibular synostosis at the top and centering the talus under the fibula with talofibular synostosis. Good results with this technique have been published [14,15]. However, bone lengthening techniques are complex, lengthy programs that require instrumentations that are not always available. They are also very time-consuming and require excellent cooperation from families, conditions that are not always present. If these conditions are not met and the environment is not conducive, it is preferable to avoid referring to protocols that are difficult or impossible to implement. In these cases, the shortening of the lower leg segment can be compensated by maintaining the equinismus of the foot to allow digitigrade support on the ground. While this does not allow a normal gait, independent walking can be achieved. These procedures do not require any specific equipment other than fine instruments. They are performed under hemostasis using a tourniquet, making the procedure relatively noninvasive. However, tibialization of the fibula with equinus foot preservation is not possible in all cases. The foot must not be malformed to allow correct digitigrade support. An upper tibial epiphysis must also be present. This eliminates cases of Kalamchi type I aplasia because, without an upper tibial epiphysis, the fibula can only be transposed under the femoral condyles. It is the Brown procedure [2,3] that never results in a stable knee [1,13]. Furthermore, the knee flexure present in type I is very difficult to correct and is often recurring. Satisfactory results after Brown surgery have been obtained in most of cases after knee disarticulation and prosthesis [4,17]. Tibialization should therefore be reserved for Kalamchi type II cases that have a functional upper end of the tibia and functional quadriceps. To enable the child to walk at a normal age, tibialization of the fibula should be performed early, ideally around one year of age. At this age, the cartilaginous model is still prominent, and during the first stage of surgery (low tibialization of the fibula), surfaces brought into contact are more cartlaginous than osseous. However, the foot is stable under the fibula after the pin is removed, and the presence of cartilage seems to allow the fibula to continue growing.

Finally, the shortening at the end of growth should not exceed 10 cm, or 15 cm at most, as beyond this point, equinus cannot compensate for the inequality. Hechard and Carlioz [8] demonstrated that, in cases of congenital malformation, the percentage of growth inhibition remains consistent throughout growth. Therefore, by calculating the percentage of shortening at birth, one can predict the extent of the inequality at bone maturity. However, in cases of bilateral tibial aplasia, which occurs in 25% of cases, the problem of length inequality does not arise, and bilateral tibialization of the fibula can be recommended.

## Conclusion

Tibial hemimelia, or congenital aplasia of the tibia, is a disabling malformation that is difficult to treat. Without treatment, children move around on all fours during the first few years and then stand with the aid of crutches. When a tibial bud is present, conservative treatment by tibialization of the fibula with compensation for the shortening of the lower leg segment by maintaining foot equinus can be proposed. This treatment is simple and can be performed in basic conditions. Although it does not allow normal foot roll, it enables the child to walk independently without assistance or crutches. Although this technique is very different from modern techniques, it offers the advantage of overcoming cultural constraints and reducing the high costs of lengthening surgery or prosthetic devices in developing countries.

## Parental consent

The surgical technique used is not innovative in that it has already been described in the literature. As a result, the opinion of an ethics committee was not sought. The parents were informed of the surgical protocol and the need for several operations.

## Funding

This work did not receive any funding.

## Author contributions

AY: revision of files and proofreading of text MO: article writing

## Conflicts of interest

The authors declare no conflicts of interest.
